# The Experience of Sleep Problems and Their Treatment in Young People at Ultra-High Risk of Psychosis: A Thematic Analysis

**DOI:** 10.3389/fpsyt.2018.00375

**Published:** 2018-08-24

**Authors:** Felicity Waite, Jonathan Bradley, Eleanor Chadwick, Sarah Reeve, Jessica C. Bird, Daniel Freeman

**Affiliations:** ^1^Department of Psychiatry, University of Oxford, Oxford, United Kingdom; ^2^Sleep and Circadian Neuroscience Institute, University of Oxford, Oxford, United Kingdom; ^3^Oxford Health NHS Foundation Trust, Oxford, United Kingdom

**Keywords:** at-risk-mental-state, ARMS, CBT, intervention, qualitative, schizophrenia

## Abstract

We view sleep disruption as a contributory causal factor in the development of psychotic experiences. Clinical trials indicate that psychological interventions targeting insomnia result in improvements in both sleep and psychotic experiences. The aim of this study was to gain the perspective of young people at ultra-high risk of psychosis on their sleep problems and associated psychological treatment. Interviews were conducted with 11 patients, aged 15–22 years, at ultra-high risk of psychosis who had received a psychological sleep intervention. Responses were analyzed using thematic analysis. Disrupted sleep timing and a lack of routine were the characteristic hallmarks of participants' sleep problems. Sleep disturbance, psychological wellbeing, and functioning had a reciprocal relationship. There were negative expectations prior to therapy, however meaningful improvements occurred in sleep, mood, and functioning. The active implementation of therapy techniques was highlighted as important. These findings indicate that the treatment of sleep problems is highly valued and has a meaningful impact on wellbeing in young people at ultra-high risk of psychosis.

## Introduction

Sleep disturbance has been identified as a potential causal factor in a range of severe mental health problems (1), including psychotic experiences (2, 3). In adolescents, sleep disruption and psychotic experiences overlap in genetic and environmental causes (4). Sleep problems are common during adolescence and have a significant impact on functioning (5, 6). In young people at high risk of serious mental health problems, sleep disturbance and circadian rhythm disruption are associated with poor outcomes and persistence of psychotic experiences (7–9). A consensus is emerging that “early treatment of sleep problems might reduce the risk of developing mental health problems and can be considered a helpful preventive strategy” (10). Sleep problems are important to be treated in their own right but also, if successfully reduced, have the potential for broad health benefits.

Cognitive Behavioral Therapy for insomnia (CBTi) has demonstrated treatment effects in adults and is the recommended first line treatment in clinical guidance (11). Importantly, improving sleep has been shown to result in additional benefits on mental health outcomes including anxiety, depression, psychotic experiences, and psychological wellbeing (12–14). However, CBTi has not been adequately tested in young people, either for its effects on sleep disturbance or potential wider effects on mental health outcomes.

Our group have conducted the first investigation of psychological interventions to treat sleep problems in young people at ultra-high risk of psychosis (15). In this case series, we found that the intervention was acceptable and may be associated with clinical benefits: following treatment, we found improvements in sleep, negative affect, and psychotic experiences. The effect sizes were large and the changes were maintained at the 1 month follow up.

Throughout the treatment development process, it is essential to incorporate the patient perspective on the phenomenology of the problem and the experience of receiving treatment. Previous qualitative studies have explored sleep problems in the absence of other mental health problems (16), or specifically in patients with sleep disturbance in the context of severe and enduring psychosis (17, 18). However, these accounts do not explore the specific developmental context of adolescence and early adulthood nor the novel target of addressing sleep as a preventative intervention for those at risk of serious mental health problems. The aim of the current study is to explore the experience of sleep problems and their treatment in young people at ultra-high risk of psychosis.

## Materials and methods

### Participants

Eleven patients, assessed as meeting criteria for ultra-high risk of psychosis based on attenuated psychosis [see (19) for full criteria] on the Comprehensive Assessment of At-Risk Mental States (CAARMS), took part in this qualitative study. All patients had recently received an adapted cognitive-behavioral intervention for sleep disturbance as part of a feasibility case series (15). Patients were identified based on their participation in the feasibility case series. All 11 patients approached consented to participate in the study. Participants were aged 15–22 years (mean = 18.27, *SD* = 1.95). Participants were employed full time (*n* = 2), part time (*n* = 2), or in education at school (*n* = 4) or university/higher education institution (*n* = 3). See Table 1 for clinical and demographic details.

**Table 1:** Demographic and clinical details of the participants, including data from baseline assessment.

**Participant number**	**Gender**	**Ethnicity**	**Number of treatment sessions**	**Insomnia (ISI)**	**Wellbeing(WEMSBS)**	**Unusual thought content (CAARMS)^a^**	**Non-bizarre ideas (CAARMS)^a^**	**Perceptual abnormalities (CAARMS)^a^**
1	Female	White	7	18	33	No	Yes	Yes
2	Male	Asian	8	18	26	Yes	Yes	Yes
3	Female	White	7	18	30	Yes	Yes	Yes
4	Male	White	8	16	36	Yes	Yes	Yes
5	Male	White	8	20	21	No	No	Yes
6	Male	White	7	14	43	Yes	No	Yes
7	Female	White	8	14	38	Yes	No	Yes
8	Female	White	7	19	38	Yes	No	No
9	Female	White	8	18	41	No	Yes	Yes
10	Male	White	8	15	49	Yes	Yes	Yes
11	Female	White	8	19	34	No	No	Yes

*Insomnia Severity Index (20); WEMWBS, Warwick and Edinburgh Mental Wellbeing Scale (21); CAARMS, Comprehensive Assessment of At Risk Mental States (19)*;

a * Score of 3+ on CAARMS subscale*.

### Intervention

The intervention is designed for young people to precisely target the key mechanisms which underpin sleep disturbance. It utilizes CBTi techniques, strategies to reduce hyperarousal, and circadian entrainment. The intervention is manualized in a modular format. All patients received 5 core modules:

1. Psychoeducation, assessment, formulation, and goal setting,
2. Establishing the environmental and lifestyle context for sleep (sleep hygiene),
3. Stimulus control (re-associating bed with sleep) and strategies to reduce hyperarousal,
4. Circadian entrainment (to regulate the timing of sleep by setting the sleep window, boosting zeitgebers for example meal and activity times, and increasing daytime activity),
5. Relapse prevention.

Additional modules (addressing nightmares, voices, and motivation) were delivered according to the individualized formulation of the young person's sleep problems. Further details of the intervention are reported by (15).

### Procedure

Ethical approval for this study was obtained from an NHS research ethics committee. The interviews were conducted at participants' homes or at their local clinic. The interviews were conducted and audio-recorded by EC, a research worker, and transcribed verbatim. The mean duration of the interviews was 23 min (*SD* = 10.8) with a range of 10–47 min.

### Semi-structured interview

A semi-structured interview was developed by FW and JB. It focused on participants' experience of sleep problems and their treatment. The interview schedule was used flexibly, with additional verbal and non-verbal cues given to encourage elaboration. The participants' own vocabulary was used in relation to psychotic-like experiences. The interview schedule included four core questions: (1) “Can you tell me a bit about how things are for you at the moment?” (2) “Have there been any changes?” (3) “What was it like for you taking part in this therapy?” (4) “Is there anything that we haven't asked that you feel may have been important to your experience?” All participants focused their answers on sleep and its improvement therefore additional targeted prompts were not necessary.

### Analysis

**Figure 1:**
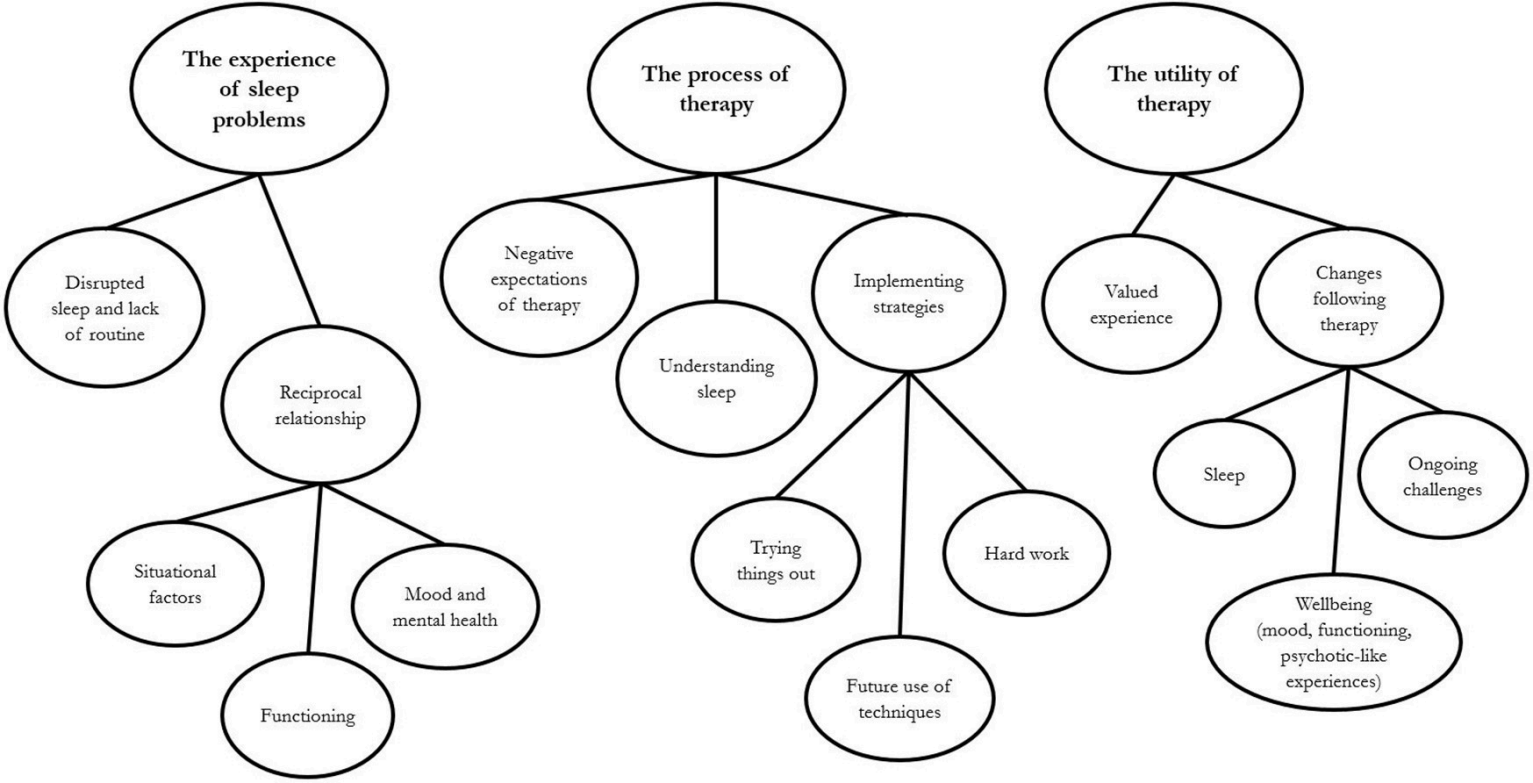
Map of thematic analysis.

Thematic analysis is a widely used method for organizing, encoding, and identifying patterns within qualitative data. Analysis of this data set was carried out following the guidance provided by Braun and Clarke (22). First, the transcripts were read whilst listening to the corresponding audiotape to ensure accuracy and familiarity with the data set. Initial codes were then applied manually to segments of data and collated. Candidate themes were identified by organizing codes manually into theme-piles and a draft thematic map was created. Candidate themes were reviewed and data extracts within the themes were checked to ensure coherence within the theme and, if necessary, themes were adjusted. A candidate thematic map was developed and reviewed to ensure it represented the data set. The map was adjusted where necessary to better fit the data set. Finally, the accepted themes were refined and a thematic map finalized for the report (see Figure 1). Initial codes and candidate themes were developed by JB. SR and JCB (who were independent of the therapy and interviews) then joined JB in reviewing and refining the themes through an iterative process until a consensus was reached on the final thematic map.

## Results

Three main themes were developed, each with sub-themes. The finalized thematic map is shown in Figure 1. The first theme centered on the experience of sleep problems; the second and third themes related to the experience and utility of the intervention.

### Theme 1: the experience of sleep problems

Participants described a strong connection between disrupted sleep timing, lack of routine, and sleep problems. A reciprocal relationship between sleep disturbance, mental health problems, and daily functioning was also described.

#### “It was a lot of just feeling tired at the wrong time” (P2) – disrupted sleep timing and lack of routine

Participants frequently described their sleep problems as being characterized by delayed sleep phase and lack of routine: “I could be like staying up to about two, three o'clock in the morning and then going to sleep, and then I'd be napping for like an hour or two during the day, and then staying up all night because I'd slept during the day” (P1). Circadian rhythm disruption included day-night reversal: “I was staying up until about like four in the morning, awake, and then I'd sleep until about three in the afternoon” (P7); “basically, I was nocturnal” (P5). This delayed sleep phase was reinforced by occupation in other activities at night: “when I went to bed, I'd sit there for ages, just on my phone or listening to music or something, without trying to sleep” (P6); “I'm ready to clock off, but my brain's not, and then I'm thinking through things” (P10); “I used to use my bed for work and things like that” (P11). Others identified a general lack of structure or predictability to their sleep patterns: “I just wouldn't really have a lot of structure to, kind of like, my sleeping pattern” (P9); “I never went to bed at the same time. I'd always be varying times, so that… Sometimes, it was even 3 or 4 h' difference” (P2).

#### “It makes everything a lot worse” (P2) – the reciprocal relationship

A complex interrelationship between sleep problems, mood, and functioning was described: “I would just sleep through the day and that like put a massive strain on like my social aspect and mood and my mental health, and I just think that it just turned my life like the other way around, so I would be up at night and then, because I was up at night, I wouldn't be up in the day and I wouldn't go out and see people because I was sleeping, and it was just… I just think it was very unhealthy” (P7). It was noted that poor sleep lowered mood, whether directly due to symptoms of insomnia, “not being able to get to sleep, not being able to stay asleep, and then being too tired during the day to actually do anything, which was really, really affecting my mood” (P3), or because of the social and functional consequences “It wasn't good for like my mental state, just like being by myself like not really talking to anyone” (P7). Others identified effects on anxiety, “it will pick up when I'm tired” (P11) or irritability “I'd just want to go home [and want to] go to sleep, and then, during the night, [I'd get] grouchy because I'd be like “I've got to go to sleep and I can't!” (P1). Participants also noticed the reverse effect, where low mood or anxiety disrupted sleep: “I'm generally feeling quite low, so that very much affects my sleep” (P2); “I'm thinking about so much that I can't relax, and that will last for like a good hour or so every night” (P10). Participants described the reciprocal relationship between sleep disruption and everyday functioning: “I just would have been in bed, doing nothing, nothing like proactive or anything” (P7); “The more I get up in the night, the more I will just stay in bed for longer” (P1).

Other situational factors were identified in the onset or maintenance of sleep problems: “when I started, I wasn't sure whether we were moving out of the area, we were staying, we were kind of up in arms and everything” (P11); “like you have hormone cycles that affect your sleep as well” (P3); “I'm traveling 3 h a day by bus and things like that, which is always quite grueling” (P10).

### Theme 2: the process of therapy

A theme was identified on the process of treating sleep problems. There were negative expectations of the therapy and its potential efficacy. Participants described two phases of therapy: understanding sleep and enacting strategies to improve it.

#### “I didn't think it would work at first” (P8) – negative expectations

There were a range of negative expectations regarding therapy. These included concerns about the potential efficacy of the treatment especially in the limited timeframe of the intervention, “when I started it, I was like no one can do this in 10 weeks” (P11); “I just thought… I'd give it a go and it probably wouldn't work” (P8). Particular intervention techniques also raised doubts: “some people [I'd heard in class], they were just like, oh, just sleep late, just, you know, like hot chocolate, or just count some sheep, and I was like, “Oh, it's just going to be that all over again!” (P1); “little methods, such as like writing things down, and how actually when you think about that, that seems like it's not really going to do anything” (P2). There were negative expectations of the specific treatment model, “I never wanted to go through CBT, ever” (P11), and the style of therapy, “my worry was it was just going to be like some depressing… fest [laughing]” (P10). For some, there were concerns about judgment from the therapist: “I was scared that I was going to kind of be judged for like my sleep and stuff” (P9).

#### “I learnt a lot about sleep” (P6) – understanding sleep

Participants' described the impact of monitoring sleep: “it sort of opened… well, not opened my eyes as such, but gave me a wider understanding of what actually affects my sleep” (P4); “with the sleep diaries, you thought more about your sleep and when you were sleeping” (P1); “I'd thought that maybe I wasn't getting enough sleep and I was tired during the day, but I didn't really think much more than that” (P6). For some participants, this revealed the scale of the sleep problem: “I didn't really realize how bad my sleep was” (P6); “I never would have imagined myself realizing how bad my sleep routine was” (P11). This detailed assessment allowed participants to gain awareness and knowledge regarding their sleep: “I learnt quite a lot, and it was just kind of… some obvious stuff, but it was said kind of in a way that I hadn't thought about before and I hadn't thought like how it was really affecting my sleep” (P9).

#### “I've got a repertoire of skills now” (P5) – implementing strategies

In developing a set of strategies to improve their sleep, participants identified a process of “trying things out”: “I can just go, “Right, let's try this.” If it doesn't work, I can try this. That works a bit. I can try this. That works even more” (P5); “It was just see what ones you can do, see what ones' work” (P9). This process included providing feedback on the strategy and then amending it or working on an alternative: “So, [my therapist] would give me a technique and then I'd come back and say ‘This worked for me; this didn't work for me,’ and then we could kind of work around that” (P3); “all the other things that we'd done before I felt they hadn't worked as much, and then we did the wind-down routine and it really helped” (P6).

Making changes to overcome sleep problems was challenging: “there's some days where I just want to like not move and just stay in my bed and just not do anything. It's hard to try and change that, to try and get myself motivated” (P7). Others identified specific strategies they found hard to implement: “the one thing I struggled with was kicking the laptop out my room, stop eating chocolate before bed, the amount of caffeine and things like that” (P11); “[my therapist] said if I get into bed and I don't fall asleep within 15 min, like to get back up again, like things like that, because once I'm in bed, I just don't really want to get back out of it [laughing]” (P8). For some, it was maintenance rather than implementation of strategies that was the challenge: “every time that I try to implement some of those strategies, there is a definite benefit, but it's quite difficult to maintain” (P10). However, implementation became easier over time: “it got easier, because I got used to what I was doing” (P8); “I've tried my best to sort of keep some of the routines… to where I don't even really think about them now” (P10).

A number of participants also talked about continuing to use strategies after completing therapy. Some were still actively using techniques and working toward their sleep goals: “I'm still doing the sleep plan” (P4); “I'm using a lot of the techniques that I was given through this” (P10); “I've been using the quarter-hour rule” (P9). Others reflected that they knew they could draw on the techniques if they needed to in the future and that the manual may be helpful for this: “if you begin to lose your sleep again, it's nice to be able to read back and remind yourself of what you did the last time” (P1); “if I sort of felt like I was having sleep problems again, I can go back to this and go, well, here's things I can use” (P5).

### Theme 3: the utility of therapy

Participants gave extensive feedback on the intervention and its outcomes. The reciprocal relationship between sleep disturbance, mental health problems, and daily functioning identified within the earlier theme was reinforced by participants' observations of the benefits when their sleep improved.

#### “I've quite enjoyed the therapy i've had” (P1) – valued experience

The majority of participants gave positive feedback on the overall experience of the intervention. “it was very good, very interesting as well” (P4); “I thought it was really, really helpful” (P3). The specific style of treatment sessions, for example home visits, contact between sessions and active engagement were noted as important: “if I was struggling during the week, I could just email and say, “Having a tough day–any advice?” sort of thing. That was really helpful” (P3); “you guys are going sort of fairly… above and beyond sort of… even coming to my house” (P10); “It was kind of very friendly. It wasn't like scary” (P9). The collaborative approach to setting the focus of the session was valued: “[my therapist] was very happy to work through any other problems that I was having at the same time” (P3); “It is based, like, what you do during the day, diet, social life, like taking all that into account along with your sleep” (P1).

Limitations of the intervention were identified. This includes the treatment outcomes: “they don't solve the situation, too much, but it does still help” (P10); “I would say just the stuff about the nightmares. For some, it does work, but for others, it doesn't, which I can understand but I would like to try and find something that would work for all of them [nightmares]” (P4). There were contrasting responses to the booklets used in therapy: “the booklet things, did confuse me a bit” (P8), “the booklets were a little over-simplified” (P3). Some elements of the intervention delivered as standard in every session (the sleep diary, insomnia questionnaire, and session summary) were described as “repetitive” (P3 and P5) or “tedious” (P9).

#### “Everything has kind of all eased up at the same time” (P11) – changes following therapy

Participants described improvements in their sleep following the intervention. Specific changes included: realigning circadian rhythm: “I do sleep now [laughing], at like the proper time” (P7), enhancing sleep quality: “waking up no more than twice during the night and not feeling concerned when I wake up” (P3), improving sleep onset latency and total sleep time: “I've been falling asleep quicker and spending more time asleep” (P4). Participants highlighted the importance of their sleep: “If I get a decent night's sleep, I'm not tired during the day” (P11); “I had originally thought it was more down to stuff during the day, like how much energy you had used on this activity or this activity or, again, what you're eating, but I didn't realize how much was down to sleep” (P10). Participants described issues related to their sleep which they would need to continue addressing such as increasing total sleep time “I'd say it's enough sleep, compared to where it was. It's still not a great amount” (P11) or hypnogogic/hypnopompic experiences “There was one thing that's still quite an issue [my therapist] called it hypnogogic experiences” (P10).

A number of participants directly linked improvements in their sleep to changes in mental health outcomes. The impact of improving sleep on mood and anxiety was reported: “I suffer from depression so it's not going to be high all the time, but because it's so highly linked with sleep, when I sleep better, I tend to have a better day” (P3); “the depression has been the big step. It's really eased. It's nowhere near where it used to be” (P11); “I find it also has helped my anxiety a lot. I'm able to cope with situations a lot better and kind of stay in control, which I think has come from me gaining control of my sleep a bit” (P9). Of particular relevance to a group at ultra-high risk of psychosis, improvements in sleep were also tentatively linked to improvements in sub-threshold psychotic symptoms: “since I've been sleeping better, my, visual things have like stopped” (P8); “I used to hear things quite a lot, like walking down the street, and that's subsided, almost entirely. I don't know whether that's a consequence of the work I've been doing with the psychologist [from the clinical team] or the sleep intervention or like a combination” (P3).

Mirroring the theme of the reciprocal relationship between sleep problems and broader difficulties, one participant summarized the wide effects of improving sleep: “the stuff that I normally wouldn't have done because I was physically just too tired, I find I can now have the energy to do that, those things, which makes me feel happy because I'm getting stuff done and I'm not getting stressed out at the fact that I haven't done the things that I wanted to do. It makes me feel happier” (P9). These concurrent changes in energy, mood, and functioning were noted both by individuals and their social networks: “my boyfriend in particular and then a couple of my friends, they've noticed how much more energy I have and how I've been contributing more in conversations” (P3).

## Discussion

To our knowledge, this is the first study to report the experience of sleep problems and their treatment in young people at ultra-high risk of psychosis. Participants gave vivid accounts about the importance of good sleep for mental health and day-to-day functioning. The experience of a delayed sleep phase, characteristic of the changes in sleep architecture in adolescence, was clearly described by participants. A lack of routine was identified as a key feature of sleep problems and thus a target for treatment.

A link between poor sleep, mood, sub-threshold psychotic experiences, and functioning was described. This is consistent with findings from a qualitative study of insomnia in adolescents with depression in which the negative impact of sleep disturbance on functioning was highlighted (23). The description of the reciprocal nature of sleep disturbance and mental health problems is aligned with accounts from patients with severe and enduring psychotic experiences (18). This supports theoretical models of sleep disruption as a causal factor in mental health problems (1). Importantly, in the current study, improvements in sleep were linked with improvements in mood, functioning, and psychological wellbeing.

Despite high treatment uptake rates there were negative expectations of therapy prior to taking part. It may be the case that some participants viewed their sleep problems as so intractable as to be untreatable – this attitude has been reported in patients with sleep problems in the context of established psychosis (17). Faulkner and Bee found that patients had pessimistic or neutral views on the likely effectiveness of “talking therapy” as an intervention for sleep problems, although individual components (e.g., stimulus control, increasing daytime activity) were seen as far more likely to be effective. What was notable, in the current study, was the spirit of experimentation, a willingness to try the intervention in spite of doubts about its efficacy. “Willingness to change” has been identified as a theme in other qualitative accounts of young people with sleep problems (24). It is unclear whether this disparity between expectation and engagement is specific to this age group (that is, whether adolescents are more willing to try strategies than other age groups) or whether it merely reflects the attitudes of those who express interest in research and so might be more willing to try the intervention in spite of reservations. Nevertheless, this finding may have specific implications for how this therapy may best be advertised and delivered in this group.

Two distinct phases of treatment were described: improving understanding of sleep and taking action to improve sleep. Prior to implementing new techniques, participants described the process of improving their understanding of sleep. Increasing knowledge of both good-quality and poor-quality sleep has been highlighted as an important target for identifying and treating sleep problems in adolescents (25). The current study indicates that young people at ultra-high risk of psychosis are keen to learn about sleep and value this information. In particular, participants described monitoring sleep as a route to increasing understanding. However, a limitation of the feasibility case series was the low rates of sleep diary completion (15). This reluctance to complete sleep diaries is consistent with findings from other studies, for example adolescents with depression identified sleep diaries as a barrier to treatment, regardless of the mode of delivery (23). Future research will need to addresses these discrepancies between desire to increase knowledge and the current tools for monitoring sleep to ensure treatment is both engaging and effective.

Previous research has described how young people at ultra-high risk of psychosis have concerns regarding discussing unusual experiences and thus delayed help seeking (26). It is possible that sleep problems carry less stigma than other mental health problems. In the current study, participants described gaining control of sleep as a route to increasing control of their mental health and overall wellbeing. This may indicate that sleep problems could be an accessible first treatment target. This is an important consideration in the context of the current clinical guidance to improve timely access to services for early psychosis (27).

There were limitations to the present study. This was a selected sample in the context of a feasibility case series. Therefore, there is potential bias in their experiences. For example, themes identified on the experience of sleep problems may reflect the content of the intervention. The most likely bias is in the enthusiasm for the intervention. However, the findings of the present study fit both with the established evidence base in adults that psychological treatments for sleep problems are effective and the emerging literature indicating wider benefits to mental health. Clearly the findings of this small qualitative study are not generalizable. For example, the current study is not intended to reflect the experiences of sleep problems in early adolescence: given the changing developmental markers in sleep architecture, social demands, and cognitive development the current findings are limited to the late adolescent period (the peak age of onset for psychotic experiences). However, the aim of this study was to use first person accounts to inform theoretical and clinical developments. We believe the findings presented provide a resource to achieve this aim.

## Conclusion

This study highlights the importance of addressing sleep problems in young people at ultra-high risk of psychosis, and provides insight on the motivations and reservations they have toward treatment. A number of key themes emerged related to treatment: negative expectations prior to treatment, two phases of therapy (understanding and action), and the importance of treatment style. These can be used to further develop the treatment and to optimize engagement. The findings support the promise of sleep interventions in young people, as reported in the feasibility study (15). Namely that, following therapy, participants reported improvements in their sleep, psychological wellbeing, and everyday functioning. The findings suggest that sleep interventions in this group should be given higher clinical and research priority.

## Ethics statement

This study was carried out in accordance with the recommendations of the Ethical Principles of Psychologists and Code of Conduct (28). All subjects gave written informed consent in accordance with the Declaration of Helsinki. The protocol was approved by the National Health Service South Central–Oxford A Research Ethics Committee (15/SC/0378).

## Author contributions

FW and JB developed the interview for data collection. JB and EC recruited participants for the study. JB provided the intervention. FW provided clinical supervision. EC conducted the interviews for data collection. JB, SR, and JCB conducted the analysis. DF and FW provided overall supervision. FW took the main responsibility for drafting of the manuscript. All authors contributed to manuscript revision, read and approved the submitted version.

### Conflict of interest statement

The authors declare that the research was conducted in the absence of any commercial or financial relationships that could be construed as a potential conflict of interest.
